# Identifying US County-level characteristics associated with high COVID-19 burden

**DOI:** 10.1186/s12889-021-11060-9

**Published:** 2021-05-28

**Authors:** Daniel Li, Sheila M. Gaynor, Corbin Quick, Jarvis T. Chen, Briana J. K. Stephenson, Brent A. Coull, Xihong Lin

**Affiliations:** 1 Department of Biostatistics, Harvard T.H. Chan School of Public Health, 655 Huntington Ave, Building II, Room 419, Boston, MA 02115 USA; 2 Ohio State University College of Medicine, Columbus, OH USA; 3 Department of Social and Behavioral Sciences, Harvard T.H. Chan School of Public Health, Boston, MA USA; 4 Department of Environmental Health, Harvard T.H. Chan School of Public Health, Boston, MA USA; 5 Department of Statistics, Harvard University, Cambridge, MA USA; 6 Broad Institute of MIT and Harvard, Cambridge, MA USA

**Keywords:** COVID-19, Health disparities, Resource allocation

## Abstract

**Background:**

Identifying county-level characteristics associated with high coronavirus 2019 (COVID-19) burden can help allow for data-driven, equitable allocation of public health intervention resources and reduce burdens on health care systems.

**Methods:**

Synthesizing data from various government and nonprofit institutions for all 3142 United States (US) counties, we studied county-level characteristics that were associated with cumulative and weekly case and death rates through 12/21/2020. We used generalized linear mixed models to model cumulative and weekly (40 repeated measures per county) cases and deaths. Cumulative and weekly models included state fixed effects and county-specific random effects. Weekly models additionally allowed covariate effects to vary by season and included US Census region-specific B-splines to adjust for temporal trends.

**Results:**

Rural counties, counties with more minorities and white/non-white segregation, and counties with more people with no high school diploma and with medical comorbidities were associated with higher cumulative COVID-19 case and death rates. In the spring, urban counties and counties with more minorities and white/non-white segregation were associated with increased weekly case and death rates. In the fall, rural counties were associated with larger weekly case and death rates. In the spring, summer, and fall, counties with more residents with socioeconomic disadvantage and medical comorbidities were associated greater weekly case and death rates.

**Conclusions:**

These county-level associations are based off complete data from the entire country, come from a single modeling framework that longitudinally analyzes the US COVID-19 pandemic at the county-level, and are applicable to guiding government resource allocation policies to different US counties.

**Supplementary Information:**

The online version contains supplementary material available at 10.1186/s12889-021-11060-9.

## Background

COVID-19 has had significant medical, social, and economic impacts on all communities in the United States (US), and efficient allocation of limited resources has been a constant challenge [1–8]. Concerns about access to testing and personal protective equipment arose during the beginning of the pandemic in January–February 2020, and more recent challenges in winter 2020–2021 include vaccine distribution, deployment and uptake [9–14]. The high counts of COVID-19 cases, hospitalizations, and deaths in the US further highlight the need to develop efficient resource allocation strategies. This will allow for better control of the pandemic, ensure an equitable response, and reduce burdens health care systems.

Current US resource allocation efforts typically use individual-level factors to prioritize high-risk people. For example, US national COVID-19 vaccine guidelines prioritized healthcare workers and long-term care residents first, and then certain essential workers and the elderly [15, 16]. However, studies looking at influenza and COVID-19 vaccine resource allocation strategies have found that additionally prioritizing regions in which disease activity has been the greatest can further reduce the number of deaths, possibly by even a factor of two [17, 18]. In addition to vaccine distribution, education efforts to promote confidence in and compliance with COVID-19 vaccines and outreach efforts to encourage the continued use of non-pharmaceutical interventions can help save even more lives [19, 20]. Therefore, understanding the characteristics of counties with high COVID-19 case and death rates can help ensure a more equitable distribution of resources such as vaccines and also guide community education and outreach efforts.

There are also additional advantages to performing county-level analyses. Studying county-level associations can better account for regional trends not captured by individual data, as individual geographic data are often unavailable or more difficult to obtain due to data privacy restrictions [21]. Furthermore, some counties may have an underreporting of COVID-19 cases and deaths for various reasons [22, 23], so there is value in considering county-level characteristics in addition to reported case and death rates for making policy decisions.

In this study, we use county-level demographic, race/ethnicity, socioeconomic, and medical comorbidities variables synthesized from various data sources to build regression models that i) study county-level covariate associations with COVID-19 total case and death rates and ii) explore season-varying county-level associations with COVID-19 weekly case and death rates. These identified county-level risk factors for high COVID-19 burden can be used to inform government policy making for more efficient resource allocation to counties.

## Methods

### Data sources

We obtained demographic, socioeconomic and comorbidity data from a COVID-19 GitHub repository that drew from the US Department of Agriculture, Area Health Resources Files, County Health Rankings and Roadmaps, Centers for Disease Control and Prevention, and Kaiser News Health [24]. We obtained COVID-19 county cases and deaths from 1/22/20–12/21/20 from USA Facts [25] and additional demographic data from the US Census Bureau [26]. USA Facts is a non-profit organization providing data about government tax revenues, expenditures, and outcomes [25]. Area Health Resources Files is a part of the federal government’s Health Resources & Services administrations that includes data on population characteristics, economics, hospital utilization, and more [27]. County Health Rankings & Roadmaps is a collaboration between the Robert Wood Johnson Foundation and University of Wisconsin that provides local community health data [28]. All data used in analyses are publicly available and can be found on our lab GitHub page (https://github.com/lin-lab/COVID-Health-Disparities).

### County-level variables

County-level cumulative and weekly COVID-19 cases and deaths as of 12/21/20 were directly obtained from USA Facts [29]. USA Facts aggregates data from the Centers of Disease Control and state and local public health agencies. County-level data were confirmed by referencing state and local agencies.

Demographic variables were obtained from the US Census Bureau and US Department of Agriculture and included county percentage ages 20–29 years, percentage ages 60+ years, percentage male, and metro/nonmetro status classification. US Department of Agriculture rural-urban continuum codes were grouped into three categories for the metro/nonmetro categorical variable: metro, population ≥ 1 million (code 1); metro or near metro, population 20,000 to 1 million (codes 2–4); nonmetro, population < 20,000 (codes 5–9) [30].

County-level population distribution by race/ethnicity, including Black/African American, Hispanic/Latino, American Indian/Native Alaskan, Asian, Native Hawaiian/Pacific Islander proportions, were directly obtained from 2019 US Census Bureau estimates [26]. County residential racial segregation indices of dissimilarity were obtained from County Health Rankings & Roadmaps [31]. These indices were originally calculated from data from US Census tracts from the American Community Survey 2014–2018. Counties with less than 100 Black/non-white residents had the index of dissimilarity set to be equal to 1.

Socioeconomic variables were obtained from Area Health Resource Files [27] and included average household size, percentage of individuals between 18 and 64 years old without health insurance, percentage in poverty, percentage of people aged > 25 years without a high school diploma, and percentage of people working in education/health care/social assistance.

Prevalence rates for several comorbidities were obtained from County Health Rankings & Roadmap [28]. Comorbidities included county-level percentages for: smoking, obesity, asthma, cancer, chronic obstructive pulmonary disease, diabetes, heart failure, hypertension, kidney disease, and stroke. Kaiser News provided total intensive care unit beds and nursing home beds.

Log transformations were applied to heavily skewed variables. Additional covariate information is available in Additional Table 1.

### Cumulative county rates models

We used a Poisson mixed model to model cumulative case and death counts for all 3142 US counties through 12/21/20. We included fixed effects for each state to account for state-to-state variation not explained by variables in the model (such as state testing rates)**,** a random effect for each county to account for overdispersion and county testing rates, and an offset term for log county population (2019 US Census estimates). Continuous covariates were scaled to have mean zero and standard deviation one for modeling.

To explicitly define the cumulative case rate model, assume *R*_*ij*_ is the number of reported cases and *P*_*ij*_ is the population of county *j* in state *i*. *R*_*ij*_ can be modeled by a Poisson mixed model with expected cases *λ*_*ij*_:

$$ \ln \left({\lambda}_{ij}\right)=\ln \left({P}_{ij}\right)+{\theta}_i+{\boldsymbol{X}}_{ij}^{\hbox{'}}\boldsymbol{\delta} +{e}_{ij}, $$

where *θ*_*i*_ is the state effect, ***X***_*ij*_ is a vector of covariates, and the  *e*_*ij*_ are county-specific random effects. The death and case fatality rate models follow the same structure. Total death rates modeled total deaths instead of total cases. Total case rates additionally used log total cases as an offset instead of log population size. For case fatality rate models, counties with total case counts of zero were set to one so the log offset would be defined.

Univariable models included a single covariate for *X*_*ij*_, and separate models were fit for each outcome and each covariate combination. Multivariable models included all covariates for *X*_*ij*_ and a single model was fit for each outcome. Estimates and confidence intervals for *δ* were used to explore associations between county-level covariates and COVID-19 rates.

### Weekly county rates models

Additional modeling of weekly county case, death, and case fatality rates from 3/23/20–12/21/20 (40 repeated measures per county) were performed to investigate how county-level associations varied by season. To define our weekly rate model, let *P*_*ij*_ be the population size and *R*_*ijt*_ be the reported cases in county *j* of state *i* on week *t*. Then *R*_*ijt*_ can be modeled by a Poisson mixed model with expected cases *λ*_*ijt*_:

$$ \ln \left({\lambda}_{ij t}\right)=\ln \left({P}_{ij}\right)+{\boldsymbol{X}}_{ij t}^{\hbox{'}}\boldsymbol{\delta} +S{(t)}^{\hbox{'}}{\beta}_s+{\tilde{b}}_{ij}+{b}_{ij t}, $$

where *X*_*ijt*_ is a vector of covariates including state fixed effects (not time-varying) and covariate season interactions, *δ* is a vector of regression coefficients, *S*(*t*)^′^*β*_*s*_ is a cubic spline basis for time with knots every 14 weeks (divides 40 repeated measures into three approximately equal windows) that varies by US Census region, ${\tilde{b}}_{ij}$ are independent and identically distributed (i.i.d.) county specific random effects with ${\tilde{b}}_{ij}\sim N\left(0,{\sigma}_1^2\right)$, and *b*_*ijt*_ are county specific longitudinal random effects with AR-1 correlation structure ***b***_*ij*_ i.i.d *N*(0, *V*(***σ***_2_)). Estimation and inference proceed using penalized quasi-likelihood as implemented in the “glmmpql” command from the MASS package in R [32–34].

The weekly death rate model is the same except weekly new deaths instead of weekly case counts are the outcome variable. A time-invariant population size offset is still used. For the weekly case fatality rate model, due to delays between COVID-19 diagnosis and official recording of death, we explored using a one-week and three-week lag for weekly COVID-19 deaths. To illustrate, for the one-week lag, weekly deaths on 12/21/20 were shifted back to have an offset for weekly cases on 12/14/20. Exploratory analyses suggested a one-week lag would roughly match national death peaks with case peaks (see results for additional information). One study had reported the median time from COVID pneumonia confirmation to death in a clinical setting was 13 days [35]. Giving an additional week for death certificate processing, we also chose to explore a three-week lag. For case fatality rate models, counties with total case counts of zero were set to one so the log offset would be defined. Additionally, if the number of weekly deaths was greater than the number of matched weekly cases, we set the weekly cases offset to be equal to the corresponding weekly deaths outcome.

Only the metro/nonmetro categorical variable and white/non-white segregation covariates from total rates modeling were used for weekly modeling. We consolidated additional variables as follows. The variable non-white percentage was directly obtained from the US Census Bureau. A composite socioeconomic disadvantage variable was calculated as the mean of the no health insurance, poverty, and no high school diploma variables. A composite comorbidities variable was calculated as the mean of the smoking, obesity, asthma, cancer, chronic obstructive pulmonary disease, diabetes, heart failure, hypertension, kidney disease, and stroke variables. Multivariable models included all covariates and their interactions with season for *X*_*ijt*_ and a single model was fit for each outcome. Estimates and confidence intervals for linear contrasts of *δ* were used to explore season-varying associations between county-level covariates and COVID-19 rates.

### Data analysis

All analyses were conducted in R. The following packages were used in formatting data: data.table, dplyr. The following packages were used in formatting results and creating plots: ggplot2, usmap, gridExtra, tidyverse, plyr. The following packages were used in modeling: glmnet, geepack, geeM, lme4, splines, MASS, glmmpql. Code for these analyses is available as described in the code availability section.

## Results

Associations of county-level characteristics with cumulative case and death rates.

**Fig. 1:**
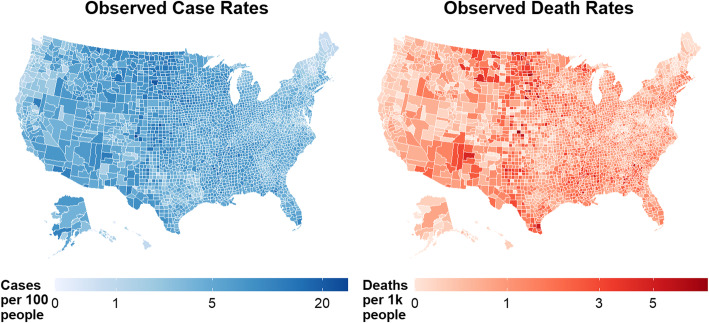
Observed total case and death rates. Observed cumulative case and death rates through 12/21/2020 for all 3142 US counties

There is substantial heterogeneity in total case and death rates among counties across the US. Figure 1 shows heatmaps of observed total case and death rates for all 3142 US counties as of 12/21/2020. Cumulative case and death rates tended to be highest among Midwestern and Southern states.

**Fig. 2:**
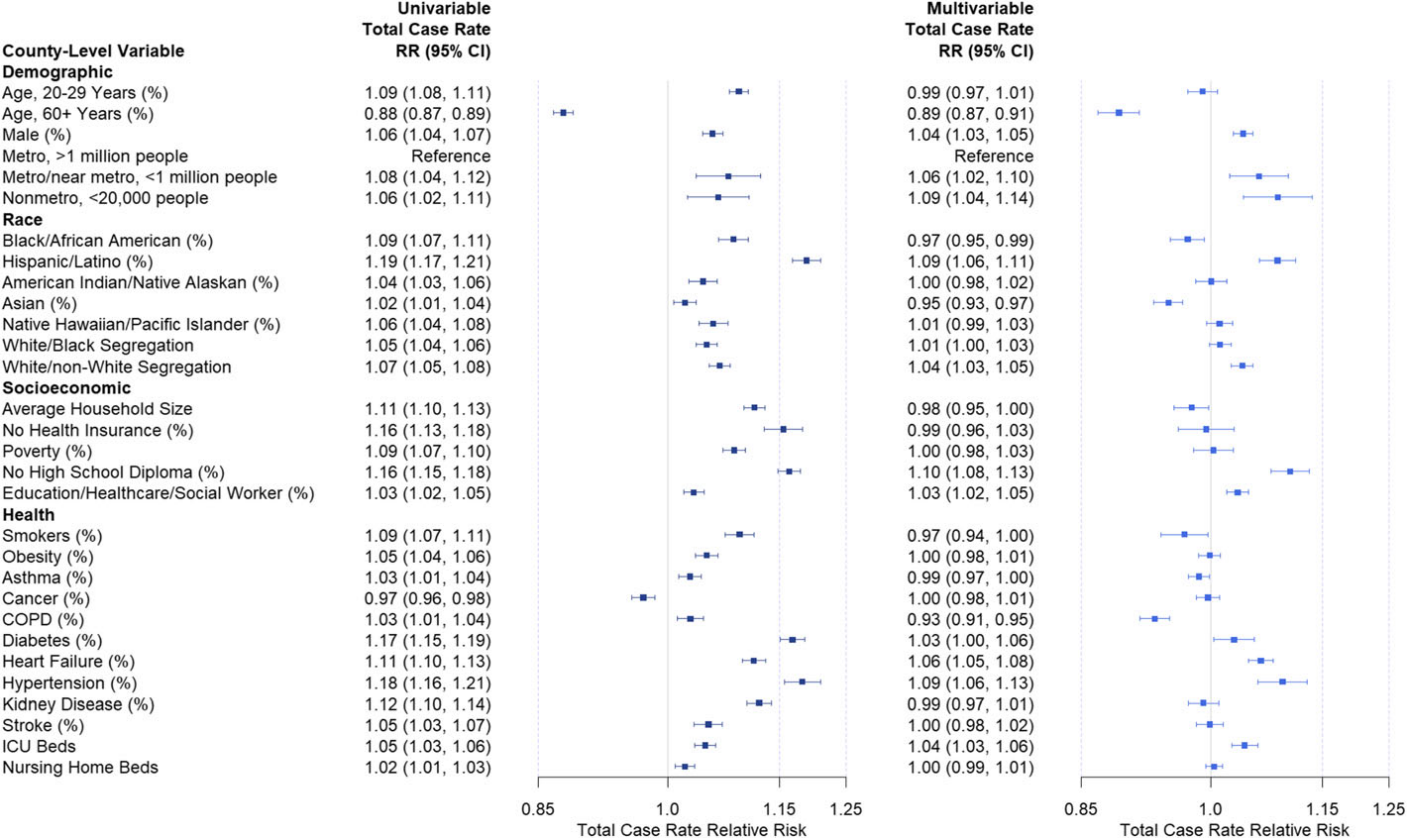
Univariable and multivariable case rate relative risks. Univariable and multivariable relative risks of demographic, socioeconomic, and health comorbidity factors on cumulative COVID-19 case rates through 12/21/20 additionally adjust for state fixed effects and county random effects. Boxes are point estimates and error bars mark 95% confidence intervals. Relative risks are for a one standard deviation increase in a variable, except for the metro/nonmetro categorical variable. COPD – chronic obstructive pulmonary disease, ICU – intensive care unit

**Fig. 3:**
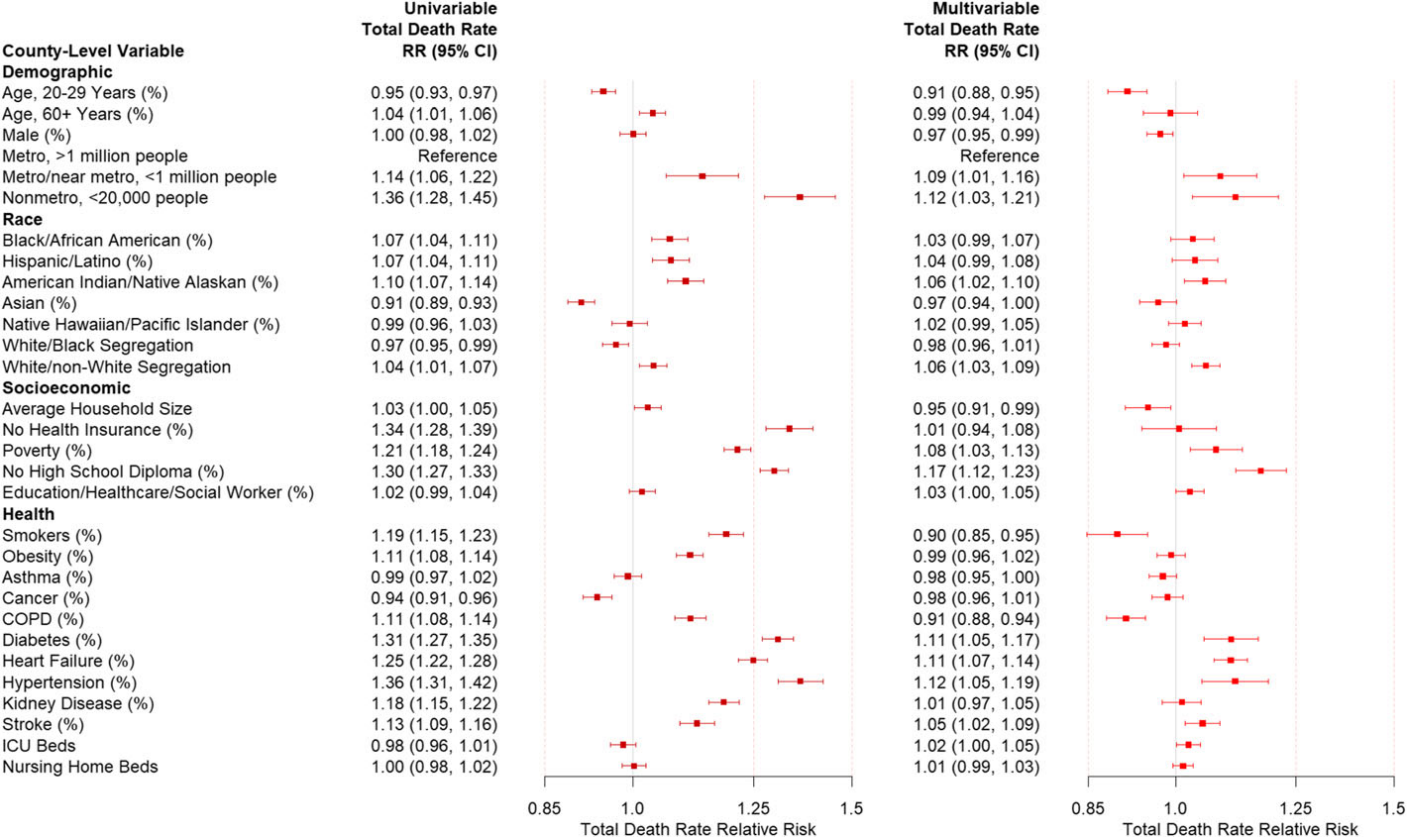
Univariable and multivariable death rate relative risks. Univariable and multivariable relative risks of demographic, socioeconomic, and health comorbidity factors on cumulative COVID-19 death rates through 12/21/20 additionally adjust for state fixed effects and county random effects. Boxes are point estimates and error bars mark 95% confidence intervals. Relative risks are for a one standard deviation increase in a variable, except for the metro/nonmetro categorical variable. COPD – chronic obstructive pulmonary disease, ICU – intensive care unit

Associational relative risks for the multiplicative increase in county COVID-19 cumulative case, death, and case fatality rates were calculated for a one standard deviation increase of a county-level variable. Figure 2 shows univariable and multivariable case rate RR (relative risk). Figure 3 shows univariable and multivariable death rate RR. Univariable analyses show that more rural counties and counties with more racial minorities, racial segregation, socioeconomic disadvantage, and increased health comorbidities tend to have higher total case and death rates. After adjustment in multivariable analyses, more rural counties, and counties with increased white/non-white segregation, poverty, no high school diplomas, diabetes, heart failure, and hypertension tended to have higher total case and/or death rates.

Many variables that were statistically significant in univariable analysis were not in multivariable analysis. Additional Fig. 1 plots Spearman correlations for all covariates and shows how many of these variables are correlated. More rural counties tend to have more males residents, residents 60+ years, and fewer minorities. Black/African American percentage tended to be positively correlated with socioeconomic and health comorbidity variables. Socioeconomic and health comorbidity variables tended to be positively correlated. Spatial patterns among different covariates also exist. Covariate heatmaps are in Additional Figs. 2 and 3. Black percentages, stroke, heart failure, hypertension, and kidney disease percentages are all greatest in the Southern states. Hispanic percentages are greatest along the Southwestern states. Smoking and chronic obstructive pulmonary disease percentages are elevated along the Appalachian Mountains.

We also explored including state testing rates as a covariate for modeling. However, we found this did not change any estimated associations adjusted for demographic, race/ethnicity, socioeconomic, and comorbidity variables because we already controlled for fixed state effects using state dummy variables. Adding state testing rates only changed the estimated state fixed effects through re-parametrization. Additional details are in the Additional Methods.

Season-varying associations of county-level characteristics with weekly case and death rates.

**Fig. 4:**
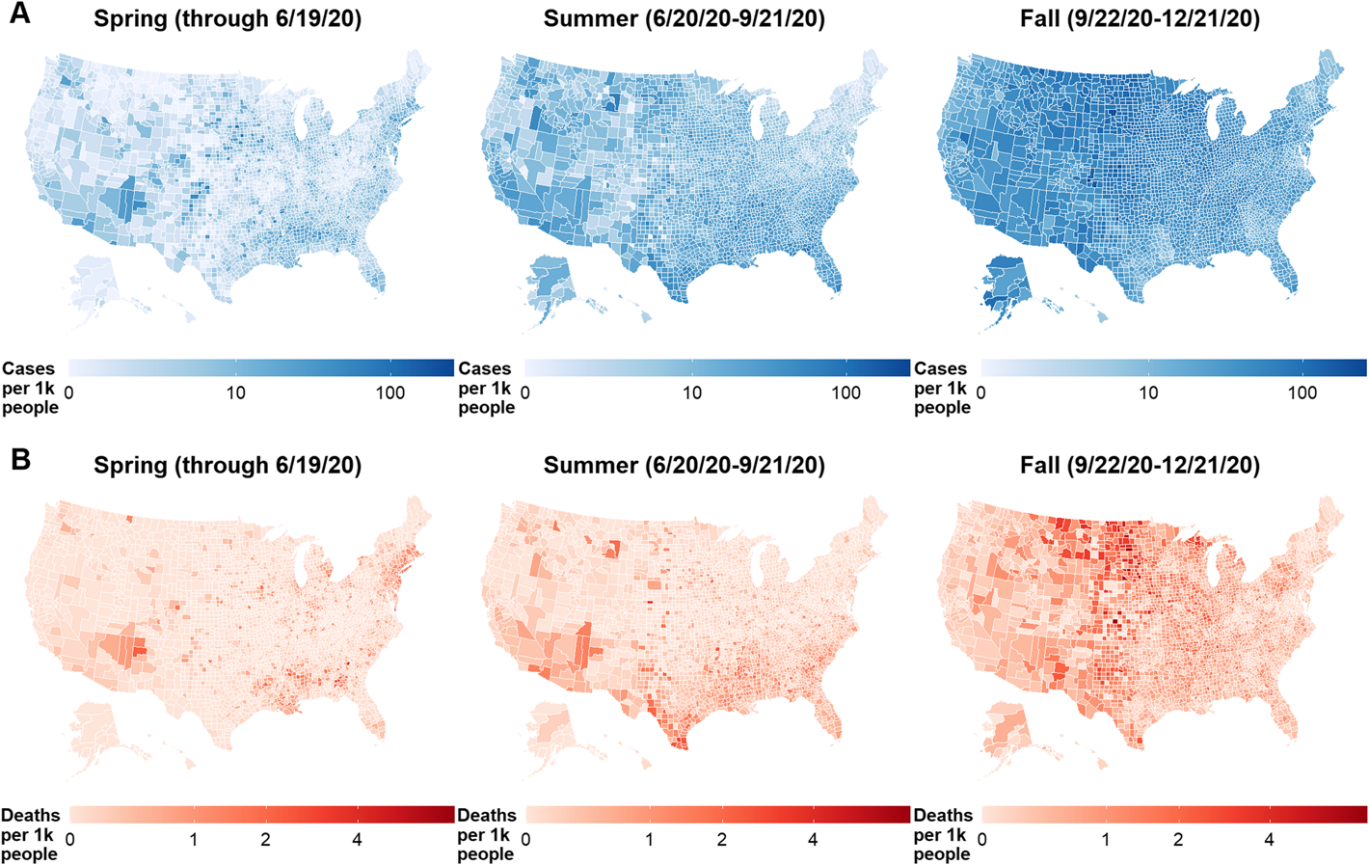
Observed case and death rates by season. Observed case and death rates by season through 12/21/2020 for all 3142 US counties

We further explored how county-level covariate associations with COVID-19 rates varied over the course of the pandemic. Figure 4 shows total case and death rates were highest in the Northeast during the spring, increased in the South during the summer, and increased everywhere including rural areas during the fall.

Longitudinal modeling of weekly county case and death rates was performed. Table 1 shows multivariable relative risks for weekly case and death rates. During the spring, more urban counties and counties with more racial minorities and racial residential segregation tended to have greater case and death rates. As the pandemic progressed, during the fall, more rural areas tended to have greater case and death rates. Socioeconomic variables such as no high school diploma and medical comorbidity variables such as hypertension were associated with increased case and death rates throughout the fall, summer, and spring.

**Table 1:** Multivariable weekly case and death rate relative risks

**Variable**	**Multivariable Weekly Case Rate Relative Risk**^a,b,c^		
	**Spring**	**Summer**	**Fall**
Metro, > 1 million people	Ref	Ref	Ref
Metro/Near Metro, < 1 million people	**0.84 (0.79, 0.88)**	**0.92 (0.88, 0.95)**	1.04 (1.00, 1.07)
Nonmetro, < 20,000 people	**0.82 (0.75, 0.90)**	**0.75 (0.71, 0.80)**	1.02 (0.98, 1.07)
Non-White (%)	**1.52 (1.46, 1.58)**	**1.04 (1.01, 1.06)**	**0.94 (0.92, 0.96)**
White/non-White Segregation	**1.06 (1.02, 1.09)**	**1.05 (1.02, 1.07)**	1.02 (1.00, 1.04)
Socioeconomic Disadvantage	**1.28 (1.23, 1.33)**	**1.26 (1.22, 1.29)**	**1.09 (1.06, 1.11)**
Comorbidities	1.00 (0.96, 1.04)	**1.06 (1.03, 1.09)**	**1.08 (1.06, 1.11)**
	**Multivariable Weekly Death Rate Relative Risk**^a,b,c^		
Metro, > 1 million people	Ref	Ref	Ref
Metro/Near Metro, < 1 million people	**0.74 (0.69, 0.79)**	**0.88 (0.83, 0.93)**	**1.40 (1.32, 1.47)**
Nonmetro, < 20,000 people	**0.76 (0.69, 0.85)**	**0.77 (0.70, 0.83)**	**1.78 (1.66, 1.91)**
Non-White (%)	**1.87 (1.79, 1.95)**	1.02 (0.98, 1.05)	**0.82 (0.80, 0.85)**
White/non-White Segregation	**1.12 (1.07, 1.16)**	**1.05 (1.01, 1.09)**	1.03 (1.00, 1.06)
Socioeconomic Disadvantage	**1.15 (1.10, 1.21)**	**1.44 (1.38, 1.49)**	**1.07 (1.03, 1.11)**
Comorbidities	**1.15 (1.09, 1.20)**	**1.23 (1.17, 1.28)**	**1.21 (1.16, 1.25)**

^a^Parentheses indicate 95% confidence intervals

^b^Bold indicates confidence interval does not contain 1

^c^Relative risks are for a one standard deviation increase in a variable, except for the metro/nonmetro categorical variable

### Associations of county-level characteristics with cumulative case fatality rates

Next, we performed exploratory total case fatality rates analyses. Assuming the ascertainment rates of reported cases vary by state or county, and by including fixed state effects and county random effects, the case fatality rate and infection fatality rate regression analyses produced identical results for covariate associations (see Additional Methods for further discussion). Observed case fatality rates did not appear to have any obvious geographic patterns (Additional Fig. 4). The case fatality rate results had similar directions to the primary death rate results, and counties with more residents age 60+ years were associated with increased case fatality rates on both univariable and multivariable analyses (Additional Fig. 5). However, these analyses are likely subject to bias due to several factors, such as differential underestimation of the total number of cases by race and ethnicity and selection bias of subjects who have been tested [22, 36].

### Season-varying associations of county-level characteristics with weekly case fatality rates

Lastly, we look at case fatality rates over time. Additional Fig. 6 shows longitudinal trends of COVID-19 case fatality rates over time. Additional Fig. 6A shows that national death rates lagged by about 1 week peak at the same time as national case rates for USAFacts data, likely due to delays between COVID-19 testing confirmation and official recording of COVID-19 deaths. Additional Fig. 6B does not show any distinct geographic case fatality rate patterns, but case fatality rates appear to be decreasing in all regions over time.

Longitudinal modeling of case rates adjusted for a one-week reporting lag (ex. weekly deaths ending on 12/21/20 were matched with weekly cases ending on 12/14/20) and three-week lag. Additional Table 2 shows multivariable season-varying weekly case fatality relative risks. Counties with more racial minorities and segregation tended to have increased case fatality rates in the spring, whereas rural counties tended to have greater case fatality rates during the fall. Counties with larger percentages of medical comorbidities tended to have increased case fatality relative risks throughout the spring, summer, and fall. Results were similar using either the one-week or three-week lag. However, just as with the cumulative case fatality rate analyses, various reporting biases exist. Additional challenges include matching individuals death and case dates using aggregated data and instability from low weekly case count offsets, especially for sparsely populated areas.

## Discussion

Using county-level data obtained from various government and nonprofit sources, we explore associations between county-level covariates and total case and death rates. We find that rural counties, counties with more white/non-white segregation, and counties with increased socioeconomic disadvantage and medical comorbidity percentages all tend to have greater cumulative case and death rates. These county-level findings are consistent with individual-level results from other studies but are more directly applicable to informing government resource allocation policies to counties. Improving public health resource distribution to vulnerable US counties can help reduce the total COVID-19 burden.

To provide a resource allocation example, these county-level associations can be used to inform state policies for optimal and equitable vaccine distribution to counties. The Center for Disease Control and Prevention’s Advisory Committee on Immunization Practices releases recommendations for vaccine distribution [15, 16], but state governments ultimately decide how to distribute vaccines and encourage vaccine uptake [37]. Because we included state fixed effects in modeling, our county-level associations are with respect to counties within a state. State government policy makers can thus consider increased allocation of vaccines and other resources to counties with greater poverty rates and lower higher school graduation rates (and other important identified features). Increased education and outreach programs may also be directed at such counties.

Our county-level association analysis results are consistent with and complement results from various individual-level studies. Racial minorities have been found to have increased COVID-19 case, hospitalization, and death rates in the spring [23, 38–41]. Older patients are more likely to develop severe COVID-19 symptoms and have greater mortality rates [42]. Household size is known to affect COVID-19 contact and transmission rates [43]. Heart failure, hypertension, and stroke are important biological and clinical risk factors for COVID-19 disease severity and mortality [42, 44]. However, county-level results are more directly applicable to guiding resource allocation policy at a county level, so there is still additional value in quantifying such associations at the county-level.

Our analyses of weekly COVID-19 case and death rates substantiate how COVID-19 has disproportionately affected various vulnerable populations at different times. The early stages of the pandemic in the spring had disproportionately affected racially diverse and socioeconomically disadvantaged urban areas. More recently in the fall, predominately white, rural, socioeconomically disadvantaged areas have been hit harder. Most research on the COVID-19 pandemic has focused on urban areas, and more studies of rural areas are needed to better characterize the experience of these diverse 46 million individuals [5, 45].

### Strengths, limitations, and future work

We are primarily interested in studying how county-level characteristics are associated with their cumulative case and death rates. These county-level associations should not be interpreted as individual level associations. Associations observed at an aggregated level may be in the same direction, different direction, or not exist at the individual level [46]. As seen in Additional Figs. 2 and 3 and noted earlier in the results section, most county-level covariates have a clear geographic pattern that likely makes estimating individual-level associations difficult. For example, counties with more residents age 60+ years were associated with lower case rates on univariable and multivariable analyses. It is possible that elderly people are more careful to avoid exposures so there might a negative association between age 60+ years and case rates at the individual-level. However, nursing homes have also been a publicly known and tragic means of COVID-19 transmission, and one might hypothesize this factor would contribute to a positive association between age 60+ years and case rates at the county level. Ecological estimates capture both individual and county-level effects, and it is difficult to separate these estimates without individual-level data [47]. Future work may additionally account for clustered exposures among vulnerable populations such as nursing home residents and prisoners.

Multiple covariates are correlated in multivariable modeling and further complicate confounding issues in estimation and inference. For example, county smoking percentage is positively associated with cumulative case and death rates on univariable analyses, but is negatively associated with both rates on multivariable analyses. Percentage smoking is highly correlated with percentage chronic obstructive pulmonary disease, diabetes, and hypertension (Spearman correlation 0.62, 0.59, and 0.51 respectively), and these strong correlations may partly contribute to the flipped association observed in multivariable analyses when all variables are simultaneously adjusted for.

For exploratory weekly case fatality rate analyses, we used a one-week and three-week lag in modeling weekly deaths. However, the delay between a reported case and death is likely to have changed across time (as treatment efficacy and government reporting infrastructure improved), and this delay may also vary between counties (different counties have different processes for reporting cases and deaths). Additional methodological research is needed to better account for this important issue.

Lastly, as with all observational studies, associational findings do not imply causality. However, the focus of our study is on identifying county-level characteristics of vulnerable counties to help guide resource allocation efforts, not estimating individual-level associations. Therefore, while very important to keep in mind, these limitations are less relevant to our primary study goals.

There are various strengths to this study. Studying county-level associations can better account for regional trends not captured by individual data, as individual geographic data are often unavailable due to data privacy restrictions [21]. Because of reporting biases in COVID-19 cases and deaths [22, 23], there is value in considering county-level characteristics in addition to reported case and death rates for making policy decisions. In the cumulative rates model, state fixed effects can account for varying reporting rates between states and county random effects can account for varying reporting rates that occur between counties (see Additional Methods for more detailed discussion). Next steps to improving analyses can include obtaining data at finer geographic resolution such as at the US census tract or zip code level. Additional variables such as neighborhood testing rates, health literacy, political polarization, and access to COVID-19 treatments are worth investigating.

## Conclusion

Multi-faceted efforts are needed to combat the pandemic and optimize COVID-19 resource allocation. Rural counties and counties with more residential racial segregation, less residents with high school diplomas, and greater prevalences of medical comorbidities have been significantly impacted by COVID-19. Such counties are important to prioritize and may require increased support. Intervention measures can include policies requiring face coverings, providing personal protective equipment to essential workers, and ensuring prioritized and robust testing, tracing, and isolation infrastructure. Outreach efforts can include vaccination and mask wearing education by engaging community leaders and health care providers. Our identified county-level associations are applicable to the entire US and can help inform equitable resource allocation, reduce burdens on health care systems, and minimize additional loss of life.

## Supplementary Information

**Additional file 1: Figure 1.** Covariate correlations. Heatmap of Spearman correlations between demographic, racial, socioeconomic, and health covariates. **Figure 2.** Demographic, racial, and socioeconomic covariate heatmaps. Demographic (yellow, aqua, blue), racial (pink, magenta, purple), and socioeconomic (red, orange) covariate heatmaps. **Figure 3.** Health covariate heatmaps. Health (white, blue, purple) covariate. **Figure 4.** Observed and estimated case, death, and case fatality rates. Observed cumulative case fatality rates through 12/21/2020 for all 3,142 US counties. **Figure 5.** Univariable and multivariable case fatality rate relative risks. Univariable and multivariable relative risks of demographic, socioeconomic, and health comorbidity factors on cumulative COVID-19 case fatality rates through 12/21/20 additionally adjust for state fixed effects and county random effects. Boxes are point estimates and error bars mark 95% confidence intervals. Relative risks are for a one standard deviation increase in a variable (see Additional Table 1), except for the metro/nonmetro categorical variable. **Figure 6.** Weekly case fatality rates. (A) Line plots of US national weekly case rates and death rates lagged by one week. Solid lines mark similar peaks between weekly case rates and lagged death rates. (B) Heatmaps of county case fatality rates by season. **Table 1.** List of county-level variables, transformations, and sources. **Table 2.** Multivariable weekly case fatality rates. Relative risks of county-level variables on weekly case fatality rates (39 repeated measurements per county) by season from 3/23/20-12/21/20 using a one-week and three-week lag for deaths. All results are from a single model that controls for state effects, US census region-specific time varying trends, and additional county overdispersion. Parentheses indicate 95% confidence intervals. Bold indicates confidence interval does not contain 1. Relative risks are for a one standard deviation increase in a variable, except for the metro/nonmetro categorical variable.

## Data Availability

The datasets analysed during the current study are available in the Lin Lab Github repository, https://github.com/lin-lab/COVID-Health-Disparities.

## Abbreviations

COVID-19: Coronavirus 2019
US: United States
