# Supplementary material for: Identifying US County-level characteristics associated with high COVID-19 burden
Source: BMC Public Health. 2021 May 28;21:1007. doi: 10.1186/s12889-021-11060-9 (PMC8162162; doi:10.1186/s12889-021-11060-9)
Supplement: Supplementary file 1 — Additional file 1: Figure 1. Covariate correlations. Heatmap of Spearman correlations between demographic, racial, socioeconomic, and health covariates. Figure 2. Demographic, racial, and socioeconomic covariate heatmaps. Demographic (yellow, aqua, blue), racial (pink, magenta, purple), and socioeconomic (red, orange) covariate heatmaps. Figure 3. Health covariate heatmaps. Health (white, blue, purple) covariate. Figure 4. Observed and estimated case, death, and case fatality rates. Observed cumulative case fatality rates through 12/21/2020 for all 3,142 US counties. Figure 5. Univariable and multivariable case fatality rate relative risks. Univariable and multivariable relative risks of demographic, socioeconomic, and health comorbidity factors on cumulative COVID-19 case fatality rates through 12/21/20 additionally adjust for state fixed effects and county random effects. Boxes are point estimates and error bars mark 95% confidence intervals. Relative risks are for a one standard deviation increase in a variable (see Additional Table 1), except for the metro/nonmetro categorical variable. Figure 6. Weekly case fatality rates. (A) Line plots of US national weekly case rates and death rates lagged by one week. Solid lines mark similar peaks between weekly case rates and lagged death rates. (B) Heatmaps of county case fatality rates by season. Table 1. List of county-level variables, transformations, and sources. Table 2. Multivariable weekly case fatality rates. Relative risks of county-level variables on weekly case fatality rates (39 repeated measurements per county) by season from 3/23/20-12/21/20 using a one-week and three-week lag for deaths. All results are from a single model that controls for state effects, US census region-specific time varying trends, and additional county overdispersion. Parentheses indicate 95% confidence intervals. Bold indicates confidence interval does not contain 1. Relative risks are for a one standard deviation increase in a variab [file 12889_2021_11060_MOESM1_ESM.pdf]

Additional Content

Additional Methods

State Reporting Rates, Case Fatality Rate, and Infection Fatality Rate Regression

We conducted case fatality rate (CFR) and infection fatality rate (IFR) regression analyses to investigate death rates among infected subjects. CFRs were calculated by dividing the number of deaths by the number of reported cases in each county. CFR regression was performed in a similar way to the cumulative death rate regression by fitting Poisson mixed models except for using an offset for log(total reported cases) instead of log(population size). IFR were calculated by dividing the number of deaths by the number of infected cases in each county.

Since county-specific number of total infected cases were not observed and would likely be underestimated by using the numbers of reported cases, we first estimated the county-specific total number of infected cases by dividing the number of total reported cases by a single constant. However, we found that though the intercept estimate had changed, the other regression coefficient estimates had not. We then allowed the reporting rates to vary by state. This time the intercept and state fixed effects had changed, but again the rest of the regression coefficient estimates were unchanged.

This motivated us to analytically explore how allowing the ascertainment rates to vary between states and counties would affect estimation of state fixed effects and the random effects parameters. To define the CFR model, assume R_{ij} is the number of reported cases and μ_{ij} is the mean number of cumulative deaths in county *j* of state *i*. Then the CFR Poisson mixed model can be written as

$$\ln(\mu_{ij}) = \ln(R_{ij}) + \theta_i + \boldsymbol{X}'_{ij}\boldsymbol{\alpha} + e_{ij},$$

where θ_i is the state fixed effect, X_{ij} is a vector of covariates, and the e_{ij} are county-specific random effects to account for overdispersion. Further suppose the e_{ij} follow a normal distribution $N(0, \sigma^2)$.

For the IFR model, let I_{ij} be the unobserved total number of infected cases in county j of state $i.$ Suppose a_{ij} is the unobserved ascertainment rate (from 0 to 1) for state *i* and county *j*. We have $I_{ij} = \frac{R_{ij}}{a_{ij}}$. Let $a_{ij} = c_i d_{ij}$, where c_i is the overall ascertainment rate for state i and d_{ij} is the multiplicative departure of the ascertainment rate of county j from the state level acertainment rate c_i. Then the IFR Poisson mixed model can be written as

$$\ln(\mu_{ij}) = \ln(I_{ij}) + \theta_i + \boldsymbol{X}'_{ij}\boldsymbol{\alpha} + e_{ij} = \ln(R_{ij}) - \ln(a_{ij}) + \theta_i + \boldsymbol{X}'_{ij}\boldsymbol{\alpha} + e_{ij}$$

$$= \ln(R_{ij}) - \ln(c_i) + \theta_i + \boldsymbol{X}'_{ij}\boldsymbol{\alpha} - \ln(d_{ij}) + b_{ij},$$

where θ_i is the state effect. Write $\gamma_i = -\ln(c_i) + \theta_i$ and $u_{ij} = -\ln(d_{ij}) + b_{ij}$. Then we have

$$\ln(\mu_{ij}) = \ln(R_{ij}) + \gamma_i + \boldsymbol{X}'_{ij}\boldsymbol{\alpha} + u_{ij}.$$

Assuming u_{ij} to be county-specific random effects following a normal distribution $N(0, \tau)$, it follows that the IFR regression model is identical to the CFR regression model except that the estimated state effects and the county-specific random effects can also be interpreted as capturing the state and county-level ascertainment rates. Therefore, the identical relative risk results for CFR and IFR regressions are presented in **Additional Figure 5**.

The CFR/IFR results differed from the cumulative death rate results possibly because of differential underestimation of asymptomatic and mildly asymptomatic cases by race/ethnicity, selection bias associated with both the subjects who were tested, e.g., symptomatic subjects were more likely to be tested and to test positive, large fluctuations in the numbers of tests from county to county, and insufficient testing capacity. Additional data collection, such as county-level testing data and race/ethnicity specific case and death counts, is needed to better estimate the number of infected cases. This will allow for more accurate of county-specific ascertainment rates and make IFR analysis results more reliable.

Varying Reporting Rates for Total Case and Death Rates

The discussion above also illustrates how varying reporting rates can be accounted for in the cumulative case and death rate models. We make this explicit below. As in the main text, assume R_{ij} is the number of reported cases and P_{ij} is the population of county *j* in state *i*. R_{ij} can be modeled by a Poisson mixed model with expected cases λ_{ij}:

$$\ln(\lambda_{ij}) = \ln(P_{ij}) + \theta_i + \boldsymbol{X}'_{ij}\boldsymbol{\alpha} + e_{ij},$$

where θ_i is the state effect, $\boldsymbol{X}_{ij}$ is a vector of covariates, $\boldsymbol{\alpha}$ is a vector of coefficients, and the e_{ij} are county-specific random effects follow a $N(0, \sigma^2)$.

From the CFR discussion, since $I_{ij} = \frac{R_{ij}}{a_{ij}}$, I_{ij} can be modeled by a Poisson mixed model with expected cases $\lambda'_{ij} = \lambda_{ij}/a_{ij}$

$$\ln(\lambda'_{ij}) = \ln(\lambda_{ij}/a_{ij}) = \ln(P_{ij}) - \ln(a_{ij}) + \theta_i + \boldsymbol{X}'_{ij}\boldsymbol{\alpha} + e_{ij}$$

$$= \ln(P_{ij}) - \ln(c_i) + \theta_i + \boldsymbol{X}'_{ij}\boldsymbol{\alpha} - \ln(d_{ij}) + b_{ij},$$

where θ_i is the state effect. Write $\gamma_i = -\ln(c_i) + \theta_i$ and $u_{ij} = -\ln(d_{ij}) + b_{ij}$. Then we have

$$\ln(\lambda'_{ij}) = \ln(R_{ij}) + \gamma_i + \boldsymbol{X}'_{ij}\boldsymbol{\alpha} + u_{ij}.$$

Assuming u_{ij} to be county-specific random effects following a normal distribution $N(0, \tau)$, it follows that the observed case rate regression for R_{ij} and true infected case rate regression for I_{ij} are the same except that the estimated state effects and the county-specific random effects can also be interpreted as capturing the state and county-level ascertainment rates.

In other words, the fixed states effects can account for any possible variation in reporting rates between states. Covariate coefficient estimates for $\boldsymbol{\alpha}$ will be unchanged, and there will be a reparameterization of the state fixed effects (from θ_i to γ_i). The county random effects account for additional variation in reporting rates between counties within a state. However, there is an additional condition that the final reparametrized random effects μ_{ij} are normally distributed. Covariate coefficient estimates for $\boldsymbol{\alpha}$ will be unchanged and there will be a reparameterization of the random effects variance (from σ^2 to τ).

To emphasize, any state-to-state variability in reporting rates can be accounted for, but only a more restricted set of within state county-to-county variability in reporting rates can be accounted for with the model as constructed. As with the CFR/IFR regression, future work can investigate allowing the county-specific ascertainment rate

to be better estimated when county-specific testing data are available. This can allow for county-to-county reporting variability to be more accurately accounted for.

Additional Fig. 1. Covariate correlations. Heatmap of Spearman correlations between demographic, racial, socioeconomic, and health covariates.

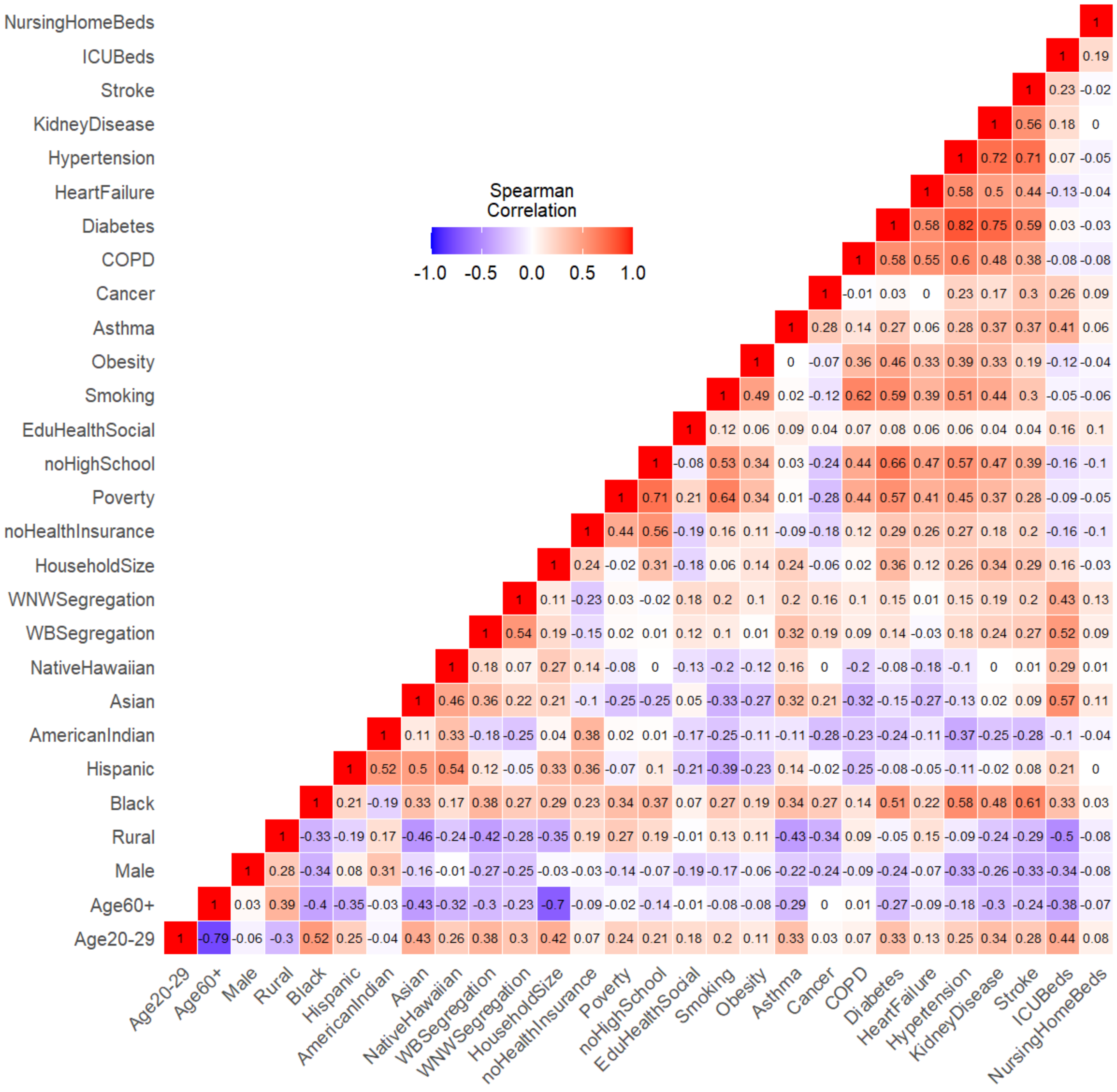

Additional Fig. 2. Demographic, racial, and socioeconomic covariate heatmaps. Demographic (yellow, aqua, blue), racial (pink, magenta, purple), and socioeconomic (red, orange) covariate heatmaps.

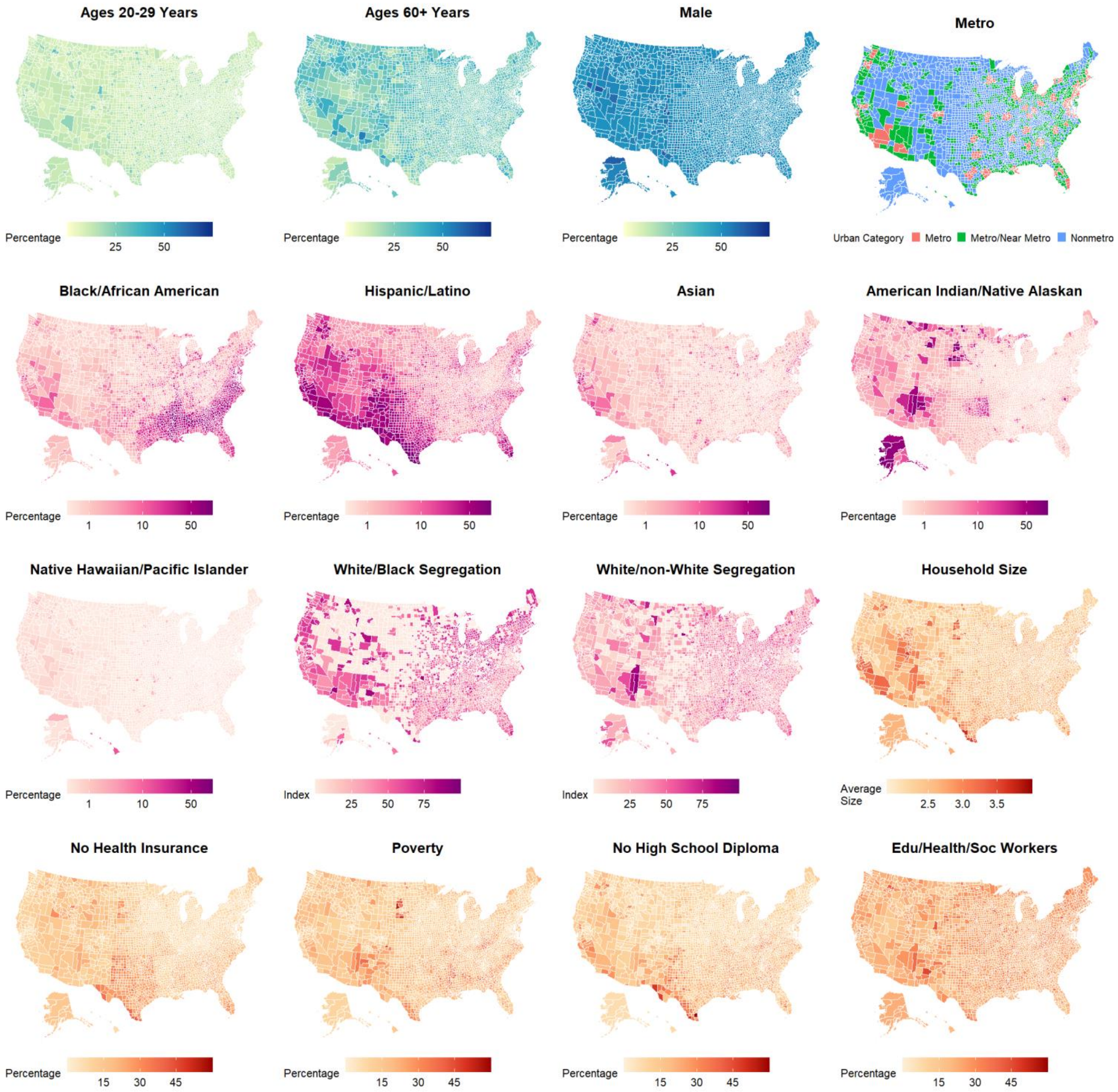

Additional Fig. 3. Health covariate heatmaps. Health (white, blue, purple) covariate

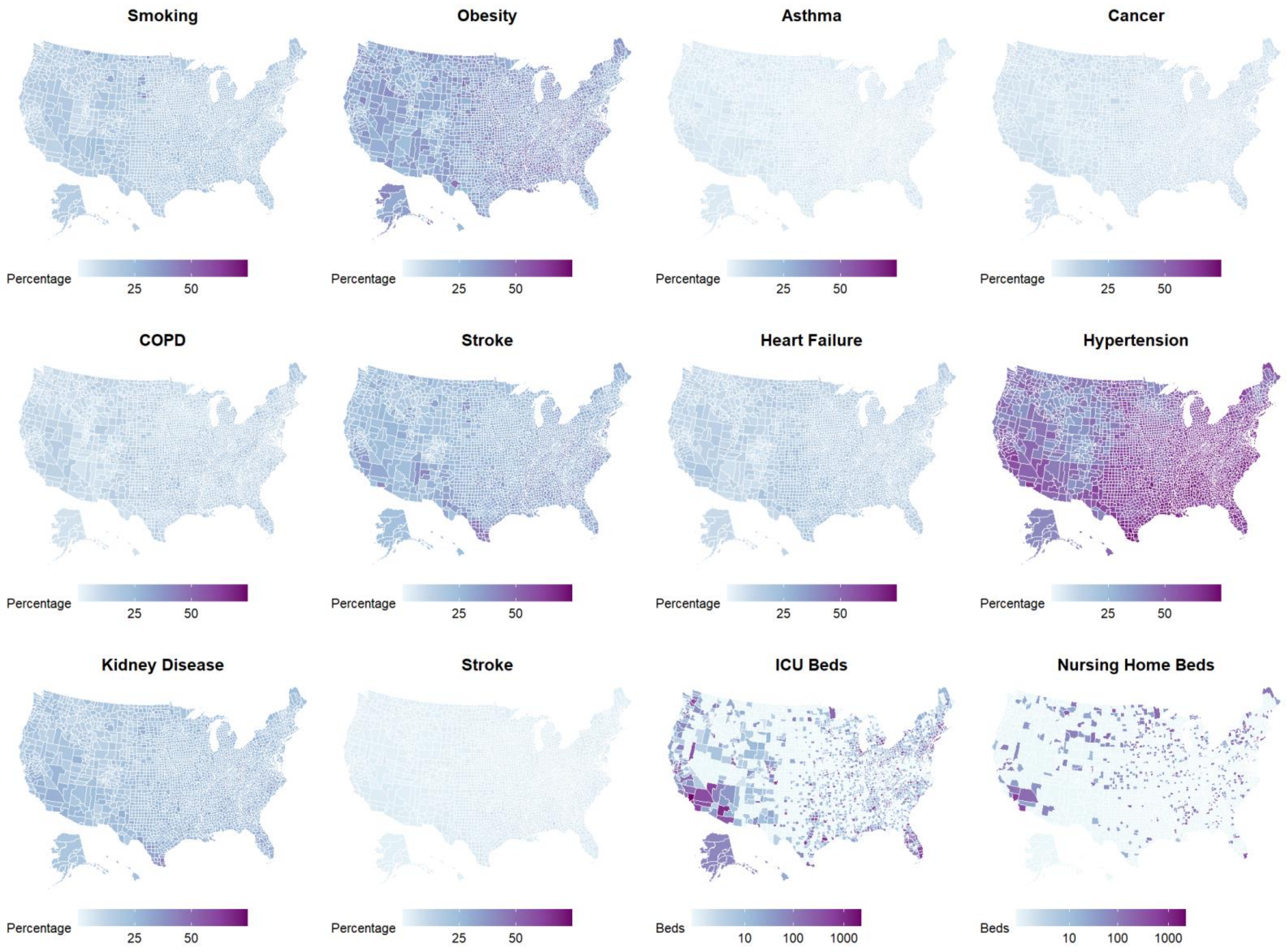

heatmaps.

Additional Fig. 4. Observed and estimated case, death, and case fatality rates. Observed cumulative case fatality rates through 12/21/2020 for all 3,142 US counties.

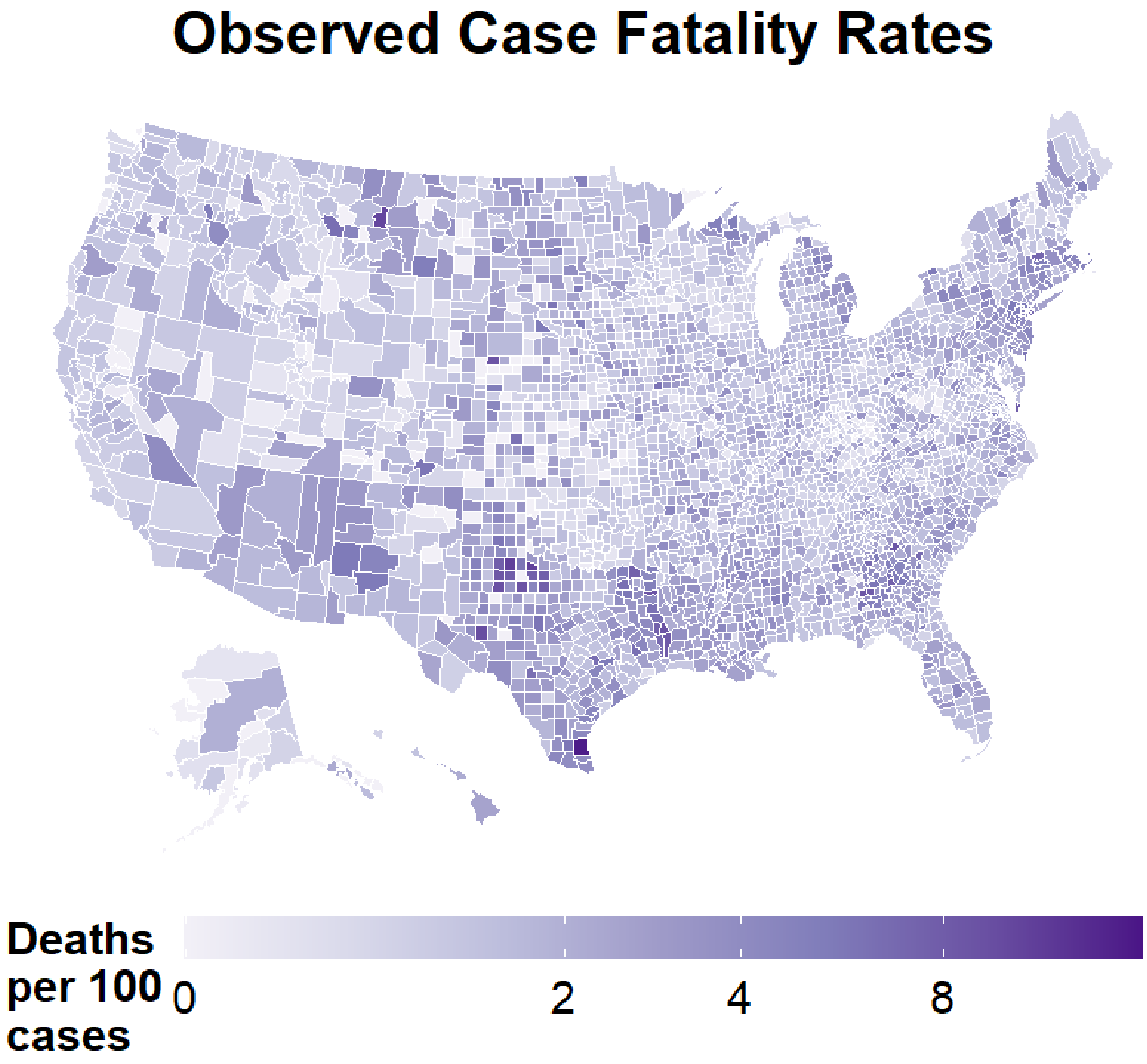

Additional Fig. 5. Univariable and multivariable case fatality rate relative risks. Univariable and multivariable relative risks of demographic, socioeconomic, and health comorbidity factors on cumulative COVID-19 case fatality rates through 12/21/20 additionally adjust for state fixed effects and county random effects. Boxes are point estimates and error bars mark 95% confidence intervals. Relative risks are for a one standard deviation increase in a variable (see **Additional Table 1**), except for the metro/nonmetro categorical variable.

County-Level Variable	Univariable Case Fatality Rate RR (95% CI)	Multivariable Case Fatality Rate RR (95% CI)
**Demographic**		
Age, 20-29 Years (%)	0.88 (0.86, 0.90)	0.93 (0.90, 0.96)
Age, 60+ Years (%)	1.17 (1.15, 1.20)	1.11 (1.07, 1.16)
Male (%)	0.94 (0.92, 0.96)	0.94 (0.92, 0.96)
Metro, >1 million people	Reference	Reference
Metro/near metro, <1 million people	1.06 (1.01, 1.13)	1.01 (0.96, 1.07)
Nonmetro, <20,000 people	1.27 (1.20, 1.34)	1.04 (0.97, 1.11)
**Race**		
Black/African American (%)	1.00 (0.97, 1.02)	1.06 (1.02, 1.10)
Hispanic/Latino (%)	0.91 (0.88, 0.94)	0.95 (0.91, 0.98)
American Indian/Native Alaskan (%)	1.06 (1.03, 1.09)	1.06 (1.03, 1.10)
Asian (%)	0.90 (0.88, 0.92)	1.00 (0.97, 1.03)
Native Hawaiian/Pacific Islander (%)	0.95 (0.92, 0.97)	1.01 (0.98, 1.04)
White/Black Segregation	0.93 (0.91, 0.95)	0.96 (0.94, 0.99)
White/non-White Segregation	0.99 (0.97, 1.01)	1.02 (0.99, 1.04)
**Socioeconomic**		
Average Household Size	0.93 (0.91, 0.95)	0.97 (0.94, 1.01)
No Health Insurance (%)	1.14 (1.10, 1.19)	1.00 (0.94, 1.07)
Poverty (%)	1.12 (1.09, 1.14)	1.07 (1.03, 1.12)
No High School Diploma (%)	1.12 (1.09, 1.14)	1.07 (1.03, 1.11)
Education/Healthcare/Social Worker (%)	1.00 (0.98, 1.02)	0.99 (0.97, 1.02)
**Health**		
Smokers (%)	1.09 (1.06, 1.12)	0.92 (0.87, 0.96)
Obesity (%)	1.06 (1.04, 1.09)	0.99 (0.97, 1.01)
Asthma (%)	0.96 (0.94, 0.98)	0.98 (0.96, 1.01)
Cancer (%)	0.97 (0.95, 0.99)	0.99 (0.97, 1.02)
COPD (%)	1.09 (1.06, 1.11)	0.98 (0.95, 1.01)
Diabetes (%)	1.13 (1.10, 1.15)	1.10 (1.05, 1.15)
Heart Failure (%)	1.13 (1.10, 1.15)	1.05 (1.02, 1.08)
Hypertension (%)	1.16 (1.12, 1.20)	1.00 (0.95, 1.06)
Kidney Disease (%)	1.06 (1.03, 1.09)	1.01 (0.98, 1.04)
Stroke (%)	1.07 (1.04, 1.10)	1.05 (1.02, 1.08)
ICU Beds	0.95 (0.93, 0.97)	1.03 (1.00, 1.06)
Nursing Home Beds	0.99 (0.97, 1.00)	0.99 (0.97, 1.01)

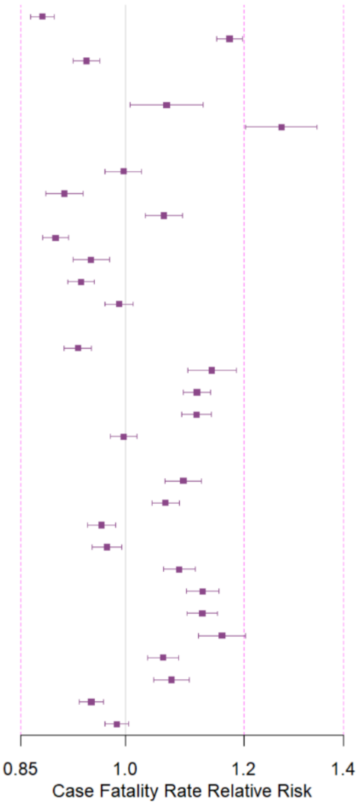

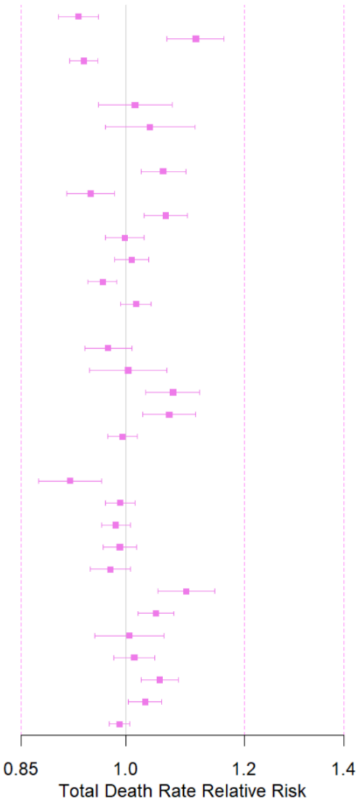

Additional Fig. 6. Weekly case fatality rates. (A) Line plots of US national weekly case rates and death rates lagged by one week. Solid lines mark similar peaks between weekly case rates and lagged death rates. (B) Heatmaps of county case fatality rates by season.

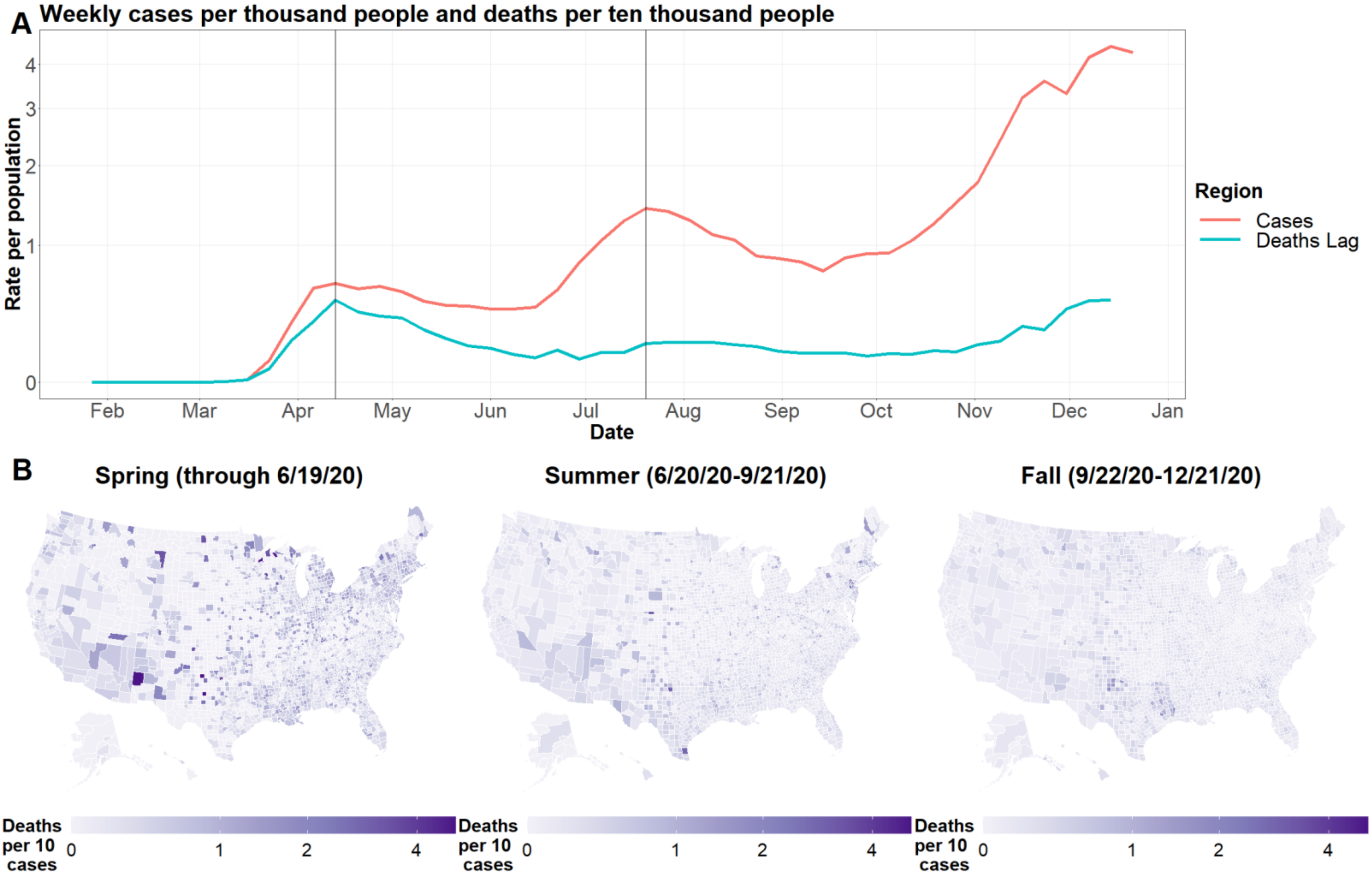

Additional Table 1. List of county-level variables, transformations, and sources.

Variable Name	Date	Transformation	Source and Description
**Outcomes**			
Cumulative COVID-19 Cases	12/21/20		USA Facts
Cumulative COVID-19 Deaths	12/21/20		USA Facts
**Demographics**			
Population Size	2019	log offset	US Census Bureau
Age, 20-29 years (%)	2019		US Census Bureau
Age, 60+ years (%)	2018		US Census Bureau
Male (%)	2018		US Census Bureau
Rural Urban Continuum Code	2013		US Department of Agriculture (2023 next scheduled update)
**Racial**			
Black/African American (%)	2019	log(x+1)	US Census Bureau
Asian (%)	2019	log(x+1)	US Census Bureau
Hispanic/Latino (%)	2019	log(x+1)	US Census Bureau
American Indian/Native Alaskan (%)	2019	log(x+1)	US Census Bureau
Native Hawaiian/Pacific Islander (%)	2019	log(x+1)	US Census Bureau
White Black Segregation Index	2014-2018		County Health Rankings & Roadmaps
White non-White Segregation Index	2014-2018		County Health Rankings & Roadmaps
**Socioeconomic**			
Average Household Size	2010		Area Health Resources Files
No Health Insurance, 18-64 years (%)	2017		Area Health Resources Files
Poverty (%)	2017		Area Health Resources Files
No High School Diploma, 25+ years (%)	2013-17		Area Health Resources Files
Education, Health Care, Social Assistance Workers (%)	2013-17		Area Health Resources Files
**Health**			
Smokers (%)	2017		County Health Rankings & Roadmaps
Obesity (%)	2017		County Health Rankings & Roadmaps
Asthma (%)	2017		County Health Rankings & Roadmaps
Cancer (%)	2017		County Health Rankings & Roadmaps
COPD (%)	2017		County Health Rankings & Roadmaps
Diabetes (%)	2017		County Health Rankings & Roadmaps

Heart Failure (%)	2017		County Health Rankings & Roadmaps
Hypertension (%)	2017		County Health Rankings & Roadmaps
Kidney Disease (%)	2017		County Health Rankings & Roadmaps
Stroke (%)	2017		County Health Rankings & Roadmaps
ICU Beds	2017	log(x+1)	Kaiser Health News
Nursing Home Beds	2017	log(x+1)	Kaiser Health News

Additional Table 2. Multivariable weekly case fatality rates. Relative risks of county-level variables on weekly case fatality rates (39 repeated measurements per county) by season from 3/23/20-12/21/20 using a one-week and three-week lag for deaths. All results are from a single model that controls for state effects, US census region-specific time varying trends, and additional county overdispersion. Parentheses indicate 95% confidence intervals. Bold indicates confidence interval does not contain 1. Relative risks are for a one standard deviation increase in a variable, except for the metro/nonmetro categorical variable.

**Variable**	**Weekly Case Fatality Rate Relative Risk**		
		**1 Week Lag**	
	**Spring**	**Summer**	**Fall**
Metro, >1 million people	Ref	Ref	Ref
Metro/Near Metro, <1 million people	0.99 (0.94, 1.05)	0.95 (0.90, 1.01)	**1.30 (1.24, 1.37)**
Nonmetro, <20,000 people	0.93 (0.85, 1.02)	1.01 (0.94, 1.09)	**1.66 (1.56, 1.77)**
Non-White (%)	**1.10 (1.05, 1.14)**	0.99 (0.96, 1.02)	**0.88 (0.86, 0.91)**
White/non-White Segregation	**1.08 (1.04, 1.12)**	1.03 (1.00, 1.06)	1.02 (1.00, 1.05)
Socioeconomic Disadvantage	**0.93 (0.90, 0.97)**	**1.08 (1.05, 1.12)**	**0.95 (0.92, 0.98)**
Comorbidities	**1.12 (1.08, 1.17)**	**1.17 (1.12, 1.21)**	**1.16 (1.12, 1.20)**
		**3 Week Lag**	
Metro, >1 million people	Ref	Ref	Ref
Metro/Near Metro, <1 million people	1.03 (0.98, 1.09)	**1.08 (1.02, 1.14)**	**1.27 (1.21, 1.34)**
Nonmetro, <20,000 people	0.94 (0.86, 1.03)	**1.21 (1.13, 1.30)**	**1.62 (1.52, 1.73)**
Non-White (%)	**1.07 (1.03, 1.11)**	**0.94 (0.91, 0.97)**	**0.88 (0.86, 0.91)**
White/non-White Segregation	**1.08 (1.04, 1.11)**	1.01 (0.98, 1.05)	1.03 (1.00, 1.06)
Socioeconomic Disadvantage	**0.90 (0.87, 0.94)**	**1.09 (1.05, 1.12)**	**0.93 (0.90, 0.96)**
Comorbidities	**1.16 (1.12, 1.21)**	**1.14 (1.10, 1.19)**	**1.21 (1.17, 1.26)**
